# Rapid determination of nematode cell and organ susceptibility to toxic treatments

**DOI:** 10.1016/j.ijpddr.2020.10.007

**Published:** 2020-10-20

**Authors:** Douglas P. Jasmer, Bruce A. Rosa, Rahul Tyagi, Makedonka Mitreva

**Affiliations:** a Department of Veterinary Microbiology and Pathology, Washington State University, Pullman, WA, 99164, USA; b Division of Infectious Diseases, Department of Medicine, Washington University School of Medicine, St. Louis, MO, 63110, USA; c Department of Genetics, Washington University School of Medicine, St. Louis, MO 63110, St. Louis, MO, 63110, USA; d McDonnell Genome Institute, Washington University School of Medicine, St. Louis, Missouri, 63108, USA

**Keywords:** Nematode, Intestine, Anthelmintics, Microscopy, Pathology, RNA-Seq

## Abstract

In research focused on the intestine of parasitic nematodes, we recently identified small molecule inhibitors toxic to intestinal cells of larval *Ascaris suum* (nematode intestinal toxins/toxicants; “NITs”). Some NITs had anthelmintic activity across the phylogenetic diversity of the Nematoda. The whole-worm motility inhibition assay quantified anthelmintic activity, but worm responses to NITs in relation to pathology or affected molecular pathways was not acquired. In this study we extended this research to more comprehensively determine in whole larval *A. suum* the cells, organ systems, molecular targets, and potential cellular pathways involved in mechanisms of toxicity leading to cell death. The experimental system utilized fluorescent nuclear probes (bisbenzimide, propidium iodide), NITs, an *A. suum* larval parasite culture system and transcriptional responses (RNA-seq) to NITs. The approach provides for rapid resolution of NIT-induced cell death among organ systems (e.g. intestine, excretory, esophagus, hypodermis and seam cells, and nervous), discriminates among NITs based on cell death profiles, and identifies cells and organ systems with the greatest NIT sensitivity (e.g. intestine and apparent neuronal cells adjacent to the nerve ring). Application was extended to identify cells and organs sensitive to several existing anthelmintics. This approach also resolved intestinal cell death and irreparable damage induced in adult *A. suum* by two NITs, establishing a new model to elucidate relevant pathologic mechanisms in adult worms. RNA-seq analysis resolved *A. suum* genes responsive to treatments with three NITs, identifying dihydroorotate dehydrogenase (uridine synthesis) and RAB GTPase(s) (vesicle transport) as potential targets/pathways leading to cell death. A set of genes induced by all three NITs tested suggest common stress or survival responses activated by NITs. Beyond the presented specific lines of research, elements of the overall experimental system presented in this study have broad application toward systematic development of new anthelmintics.

## Highlights

• A unique rapid cell death assay was developed for parasitic nematodes.
• Multiple drug-like molecules cause widespread cell death in many organs of *A. suum*.
• Multiple cell and organ systems were validated as targets for anthelmintics.
• Potential drug targets/pathways were implicated in activating cell death processes.

## Graphical abstract

**Figure:**
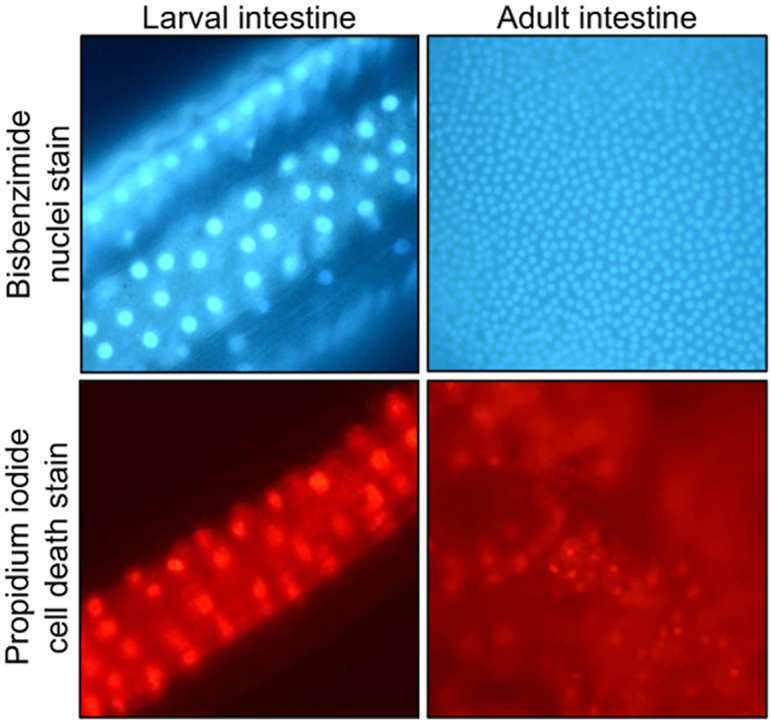

## 1 Introduction

Parasitic nematodes cause widespread, debilitating diseases that produce substantial mortality and morbidity among the global poor in human populations. These pathogens also reduce food production in livestock and plants that in turn differentially restrict nutritional resources of the global poor. Anthelmintic treatments play a major role in the control of nematode pathogens. However, the limited selection of efficacious anthelmintics and emerging pathogen resistance to anthelmintics (Sangster et al., 2018) identifies a need to expand the selection of drugs available for therapy and control of parasitic nematodes. A surge in research to increase the limited arsenal of available anthelmintic compounds has stemmed from several advances, including expanding parasite multi-omics resources, technological advances in biologic screening methods, access to expanding small molecule inhibitor libraries, and enhanced computational methods that integrate biologic data with large knowledge bases related to both drug and inhibitor compounds (examples include (Taylor et al., 2013; Tyagi et al., 2018; Tyagi et al., 2019; Jasmer et al., 2020)). These omics-driven approaches have proven quite effective in identifying small molecule inhibitors that are toxic to parasitic nematodes and have multiple applications to anthelmintic research. For instance, they may warrant development as anthelmintics, or provide much-needed research tools to dissect mechanisms relevant to drug discovery.

As one example, our research has focused on the nematode intestine due to its apparent hypersensitivity to some anthelmintics and because it has relative tractability to experimental approaches compared to other tissues (Jasmer et al., 2019). In recent progress (Jasmer et al., 2020), we identified a set of nematode intestinal toxins/toxicants (NITs) that caused apparent irreparable intestinal cell damage in *A. suum* L3 and L4. This progress was accomplished using a *de novo* approach involving intestinal multi-omics databases, coupled with pathway and drug database analysis to identify druggable targets and related small molecule inhibitors, respectively. Several NITs were also efficacious against phylogenetically diverse nematode pathogens (*Brugia pahangi* and *Trichuris muris*), indicating broader application of those findings. Although NITs include drugs approved for use in humans, and thus may represent anthelmintic candidates, they also represent valuable tools to obtain basic information underlying toxic effects, which has relevance in more general terms to anthelmintic research.

Many screens (although not all (Weeks et al., 2018)) to identify anthelmintic products rely on whole-worm motility assays to assess efficacy. While important, immotility is a non-specific outcome that may result from many different causes. The *A. suum* system provided compelling evidence that NITs can cause tissue damage inclusive of cell death, which is a specific end point with important implications for anthelmintic research. For instance, two major mechanisms of cell death dominate research in *C. elegans*, apoptosis and necrosis (Li et al., 2013), and while autophagy is often a protective response, it can contribute to the two predominant cell death pathways (Samara et al., 2008). Of importance is that each of the three processes is latent and inducible and can be pharmacologically manipulated in cells (Oh et al., 2003; Green and Kroemer, 2005; Chen et al., 2018), increasing the significance of this topic. Clear establishment of experimental treatments that can induce cell death will validate an important end point achievable in parasitic nematodes and identify tools to investigate mechanism involved. For instance, prospective cellular targets of NITs are already known. Those targets and the pathways in which they reside might, when perturbed, reflect points at which cell death processes can be initiated. Thus, those targets, other components of affected pathways and then specific components regulating apoptosis, necrosis or other cell death pathways all present prospective targets for anthelmintic disruption. Coupled with experimental treatments that induce cell death (e.g. NITs), cell pathways that respond early after treatment may inform about specific (NIT) targets and pathways that initiate progression toward cell death. This information has high value toward dissecting both pathways and mechanisms that lead to cell death processes. RNA-seq represents one of the more robust and sensitive tools to investigate gene responses to experimental treatments, and it has been used to accomplish these goals (e.g. (Alexander-Dann et al., 2018; Pabon et al., 2018; Paananen and Fortino, 2019)).

There currently are no existing methods that support rapid, comprehensive monitoring of live cells or cell death in intact parasitic nematodes, and this capability would have high value for experimental dissection of mechanisms related to this outcome. Thus, the objective of the current study was to devise an experimental approach that can illuminate all cells in live *A. suum* L3 and L4 stages with fluorescent nuclear probes (using bisbenzimide, BB) and provide a rapid resolution of cell death among organ systems conferred by NIT treatments (BB in combination with vital dye propidium iodide, PI), while comparing the performance of NITs in causing cell death among cells and organ systems (PI labeling profiles). The approach also identified cells susceptible to several existing anthelmintics, and when extended to adult *A. suum,* NIT-induced cell death was documented in freshly dissected intestine. Thus, a method was developed to inventory cell and organ system targets of any of a number of toxins/toxicants of interest in whole parasitic nematodes, while also demonstrating previously unrealized potential of many different organs as targets for anthelmintics. The pathological profiling was complemented with molecular profiles, using RNA-seq based transcriptional profiling of L3 treated individually with several NITs leading to identification of cellular pathways and targets that may represent antecedents to cell death illuminated in PI assays. The results show that the approach successfully discriminated performance among NITs in relation to their toxicity for cells and organ systems.

## 2 Methods

### 2.1 Ethics statement

All animal experiments were carried out under protocols approved by Washington State University Institutional Animal Care and Use Committee, protocol 4097. The protocols meet requirements of AVMA Guidelines for the Euthanasia of Animals: 2013 Edition; Guide for the Care and Use of Laboratory Animals: 2011 Edition, National Research Council, and USA Animal Welfare Act and Animal Welfare Regulations: 2017 Edition (AWA), US Department of Agriculture.

### 2.2 Ascaris suum L3, L4 and adult

*A. suum* lung-stage L3 were obtained as described before (Jasmer et al., 2020). Briefly, adult female *A. suum* were collected from the intestines of swine that were processed for slaughter at the University of Idaho Meat Science Laboratory (Moscow, Idaho). Eggs stripped from the last 3 cm of *A. suum* uterus were washed in PBS (phosphate buffered saline, pH 7.4) then decorticated using 0.25% hypochlorite until decortication was observed (usually within 4 min). Decorticated eggs were rinsed in 50 mL double distilled water 3 times, and eggs were then cultured to the infective stage at 20 °C for 60 days in 0.1 M H_2_SO_4_ (Oksanen et al., 1990). Larvated eggs were then washed in 50 mL distilled water 3 times and stored at 4 °C until used.

Third-stage larvae (L3) were obtained from lungs (Urban and Douvres, 1981) and trachea of New Zealand white rabbits (5.5–6.5 weeks old, Western Oregon Rabbit Company, Philomath, OR) after oral infection with 4000 larvated eggs. Intact lungs, including trachea, were dissected from euthanized rabbits at 8 days post-infection, and L3 obtained by lavage (Jasmer et al., 2020). Isolated L3 were settled by gravity and then washed in 3 sequential 50 mL volumes of warm PBS followed by 3 sequential 15 mL volumes, with intervening gravity sedimentation and discard of supernatant PBS. Extracted and cleaned larvae were then suspended in RPMI medium (R8758, Sigma-Aldrich, St. Louis MO containing 10% swine serum, 100 units penicillin and 100 μg Streptomycin/mL; P0781, Sigma-Aldrich, St. Louis MO) and then if used for testing as L3, dispensed into wells of 96-well plates (3595, Costar, Corning Inc., Corning, NY, triplicate wells for each treatment), with a total volume of 100 μL culture medium containing treatments with diluent (DMSO, 922401 JT Baker, Center Valley PA) at 1%. L3 were then incubated at 37 °C in 5% CO_2_ for times prescribed for each experimental design. Alternatively, to obtain L4, L3 were incubated in 1 mL culture medium contained in a 15 mL polypropylene tube (62.554.100, Sarstedt, Newton NC) with a loosened screw cap for 3 days, and medium was replaced daily with fresh medium. L3 molt to L4 by day 3. L4 generated in this way were dispensed into wells of 96 well plates used for experiments described here and incubated as for L3.

### 2.3 Fluorescent marker labeling

To demonstrate the ability of L3 and L4 *A. suum* to ingest fluorescent probes, two function-based fluorescent probes were used in feeding assays: 1) beta-ala,lys-AMCA (BAL-AMCA), 200 μM (BP0352, BioTrend, Zurich, Switzerland) (Meissner et al., 2004); and 2) DQ Green-BSA (D-12050, Molecular Probes, Eugene OR), 100 μg/mL (Vazquez and Colombo, 2009). L3 and L4 were cultured in the presence of these probes for 4 h prior to assessment of ingestion. Bisbenzimide (BB,; Hoechst nuclear dye 33258, Sigma-Aldrich, St. Louis MO) was used at 10 μg/mL and incubated with larvae for 4 h prior to assessment by fluorescent microscopy to document whole larva inventories of nuclei in cells and organs observable with this dye. Propidium iodide (PI, P4170, Sigma-Aldrich, St. Louis MO) was used at 100 μM, and incubated along with BB for 4 h prior to addition of experimental treatments. Preincubation was intended to maximize access of tissues to the fluorescent probes in presence of various treatments. For most experiments, larvae were then incubated with the two (BB and/or PI) fluorescent probes and the treatment for the duration of the experiment.

### 2.4 Microscopy and imaging

Fluorescent labelling was monitored using a Nikon Diaphot 300 inverted microscope equipped with epifluorescence capabilities (UV-2A filter (blue), BB, BAL-AMCA; G1A, PI; B2A, DQ-BSA) and recorded with a Nikon D5100 digital camera. Most observations were made using the 20× microscope objective (200× magnification). To better resolve cells and organs in which BB stained nuclei reside, larvae were viewed using a Nikon Optiphot compound microscope equipped with differential interference contrast (DIC) filters, epifluorescence capabilities and a Nikon D5100 digital camera. To optimize resolution of DIC/fluorescence details, images were captured in movie mode, and then selected screen shots were copied and used to produce final digital images.

### 2.5 Treatments

A set of chemicals identified as nematode intestinal toxins/toxicants (NITs) (Jasmer et al., 2020) were used in experiments here and included: alvocidib (S1230), sunitinib (S7781), Selleckchem Houston, TX; CID1067700 (SML054), leflunomide (L5025), Sigma-Aldrich, St. Louis, MO; ruxolitinib (tlrl-rux), InvivoGen, San Diego, CA; staurosporine (S-9300), LC Laboratories, Woburn, MA. Anthelmintics used in these experiments included: albendazole sulfoxide (SO) (35395), levamisole (L5796), ivermectin (I8898), Sigma-Aldrich, St. Louis MO and pyrantel tartrate (T0395), Target Mol, Wellesley Hills MA. Combretastatin A4 (C7744), Sigma-Aldrich, St. Louis MO, binds to tubulin at the colchicine binding site, similar to albendazole, was identified as toxic for *A. suum* L3 and L4 (Jasmer et al., 2020) and was used to compare effects with albendazole in this investigation. Urea (4111–01), JT Baker, Center Valley PA; and D-Sorbitol (S1876), Sigma-Aldrich, St. Louis MO were used to assess chaotropic (urea) and osmotic (urea, sorbitol) influences on PI labeling. DMSO was used as diluent for chemical treatments, except urea and sorbitol (diluted in culture media), and concentrations were adjusted to achieve a maximum 1% DMSO after all components were included for each experiment. Control wells were adjusted according to diluent used for treatment wells. With exceptions noted here, treatments were initiated at 500 μM concentration to better ensure the possibility of achieving a positive result with PI labeling based on previous experience (Jasmer et al., 2020). Exceptions to the general scenario included staurosporine (initial concentration 25 μM), levamisole (a maximum of 10 mM) because relatively high concentrations of this drug have been used to anesthetize nematodes (Lockery et al., 2012; Patel and Soto, 2013), and urea and sorbitol (1 M), to ensure initial chaotropic and osmolarity values well above physiologic levels. Dose response for selected treatments was determined for two-fold serial dilutions over a range of 5 concentrations.

### 2.6 PI labeling and PI nuclear staining profiles of *A. suum* L4

L4 larvae were used for these assays because toxic effects on L3 can induce the larvae ensheathed with deformation (LED) phenotype (Jasmer et al., 2020), which involves molting (days 2–3 in culture) and is associated with other pathologic changes that would confound interpretation of results reflected by PI labeling. In the general setup, 30–40 L4 larvae were plated per well (96 well plate) and incubated with BB and PI for 4 h prior to addition of NITs, anthelmintics and other treatments. Culture continued for 2 days prior to assessment of nuclei labeled with PI. Although labeled nuclei could be viewed without the washout of fluorescent probes, resolution was enhanced following three 2-vol washes of wells with PBS. The first 20 L4 encountered were scored for PI labeling using the 20× objective of the inverted microscope. Positive labeling required at least two nuclei showing bright fluorescence in a given L4, although control larvae were generally completely devoid of labeling. Exceptions for control and treated larvae were larvae that displayed mechanical damage (broken into pieces) from handling, and in some cases those that had failed to completely molt to L4. Both presentations showed PI labeling and were excluded from the analysis. A modest nomenclature of PI labeling patterns included “sparse” labeling of scattered nuclei, or “patchy” labeling that involved one or more groups of >5 nuclei with otherwise non-contiguous staining. The goal with this nomenclature was to capture broad categories of cells staining in organ systems while gaining a sense of variability in staining patterns, rather than to classify every permutation of labeling, which is unrealistic. PI nuclear staining profiles were determined for the 20 L4 in a treatment with each L4 scored for PI nuclear staining across categories designated by organ, specific nuclei and specified regions of larvae that display PI nuclear labeling (see results). The numbers compiled among 20 L4 for each category, and then compiled across all categories produced the PI nuclear staining profile for a treatment. PI profiles could then be compared to those obtained for control L4, among treatments, and within dilution series.

### 2.7 pH effects

Assays were conducted to control for pH effects on PI labeling independent of toxins/toxicant effects. pH of RPMI media buffered by bicarbonate and containing antibiotics was determined after addition of NITs and other treatments (at the highest concentration used for each) and then incubated at 37 °C in 5% CO_2_ for 2 h. To reduce equilibration of media with ambient atmosphere during pH measurements, wells of 96 well plates were loosely fitted with film designed to seal wells for PCR analysis (PlateMax, UC-500, Corning-Axygen, Oneonta NY). Loose fitting permitted equilibrium with incubator atmosphere. After the 2-h incubation period, and prior to removal from the chamber, the film was pressed to seal wells. Wells were maintained at 37 °C on a hot plate (PC 420D with temperature controller, Corning, Tewkesbury, MA) then a slit was created in the film over each well just prior to insertion of a pH microprobe (FiveGo pH meter with an InLab Micro Electrode, Mettler Toledo, Columbus OH). Three replicate wells were measured for each treatment and compared to media control, with and without 1% DMSO. Once a range of pH was determined for culture conditions, buffering ability was determined for RPMI media buffered by bicarbonate (R8758), Sigma-Aldrich, St. Louis MO, or HEPES (25 mM, R5886), Sigma-Aldrich, St. Louis MO, with starting pH up to 9.0. HEPES, but not bicarbonate, buffered media maintained the pH near the highest pH achieved with NITs and other treatments (see results). Next, PI labeling of L4 cultured in complete medium buffered by 25 mM HEPES with a starting pH of 8.0, 8.5 or 9.0 was assessed after incubation for 2 days at 37 °C in 5% CO_2_. Wells were run in duplicate, each containing approximately 40 larvae. PI assessment (first 20 L4) and pH measurements were made for each well using our standard methods.

### 2.8 Effects on EGCN

Area, length and width measurements were made for EGCN of L4 cultured for 2 days with staurosporine (25 μM), sunitinib (500 μM) or control treatments each containing BB to document resultant atrophy of EGCN. The first 10 L4 encountered with lateral lines facing up and down (EGCN at widest profile) were photographed under epifluorescence for identical exposure times. Area measurements on digital photographs were determined by outlining BB labeled EGCN with the drawing tool in Image J (Schneider et al., 2012) and determining the number of pixels confined within the circumscribed area. Length (anterior to posterior) and width (lateral) measurements in pixels were determine for each EGCN using the line tool in Image J. Mean area, length and width measurements were compared for EGCN from each treatment group.

### 2.9 PI labeling and PI nuclear staining profiles of adult *A. suum*

BB was used to label nuclei in adult *A. suum* using two different approaches. One involved injecting BB to achieve 10 μg/ml (1% DMSO) into the pseudocoelom and incubating for 1 h, after which nuclear labeling was assessed either directly through the cuticle (EGCN) or in dissected tissues. The volume of BB (1 mg/mL in DMSO, 1:100 dilution) for injection was based on mass measurements of adult worms. Injection of live worms depended on movement that would distribute BB throughout the body. However, after treatments described below, movement could not be relied upon for this purpose and then tissues dissected from worms were incubated in PBS with BB and PI for 30 min to label nuclei for assessment. The PI assay focused on the mid-portion of intestine that lies free in the pseudocoelom because this segment could be dissected with minimal potential tissue damage from handling. Experiments to determine effects of staurosporine (25 μM) and leflunomide (500 μM) on intestinal cells were conducted on adult *A. suum* obtained as described above from swine processed for market. Adult worms were washed extensively in PBS, 37 °C, then single adult worms were placed in 250 mL culture flasks, Falcon Brand (353084), Becton Dickinson, Franklin Lakes NJ, containing 50 mL of PBS and staurosporine or leflunomide with 1% DMSO. Control worms were maintained in PBS and 1% DMSO. Worms were cultured for 2 days at 37 °C, 5% CO_2_, and movement was recorded on each day. At the end of this treatment duration, intestinal tissue was dissected and stained with BB and PI under standard conditions, and then evaluated using light and epifluorescence capabilities of the inverted microscope. Images were captured as described for the L4.

### 2.10 RNA-seq and differential expression analysis

Freshly isolated *A. suum* L3s were treated with NITs (500 μM leflunomide, 500 μM CID1067700 and 25 μM staurosporine), collected 2 and 4 h after treatment and rinsed 3 times in ice cold phosphate buffered saline (PBS, pH 7.4). Following sedimentation in 1.5 mL microfuge tubes, pelleted larvae were overlaid with 50 μl TRIzol reagent (Invitrogen/Life Technologies, Carlsbad, CA), then stored frozen at −80 °C. RNA was extracted by homogenizing larval pellets as they thawed using a microfuge pestle, and then processing extracts according to the manufacturer's instructions. Ethanol pellets of isolated RNA were shipped to Washington University for further processing and sequencing. cDNA libraries were prepared from RNA samples using the Clontech SMARTer universal low input RNA kit to maximize yield and processed cDNA was sequenced on the Illumina NovaSeq S4 platform (paired-end 150bp reads). After adapter trimming using Trimmomatic v0.36 (Bolger et al., 2014), RNA-seq reads were aligned to the *A. suum* genome assembly (PRJNA62057. WBPS14 (Wang et al., 2017)) using the STAR aligner (Dobin et al., 2013) (v2.7.3a; 2-pass mode, basic). All raw RNA-Seq fastq files were uploaded to the NCBI Sequence Read Archive (SRA (Leinonen et al., 2011)), and complete sample metadata, read count statistics and accession information are provided in Supplementary Table S1. Each differential expression comparison of NIT vs control at a matched timepoint was performed using DESeq2 (version 1.24.0) (Anders and Huber, 2010) with default settings, and a minimum *P*-value significance threshold of 0.05 (after False Discovery Rate [FDR (Benjamini and Hochberg, 1995)] correction for the number of tests). Principal components analysis (PCA) was carried out using DESeq2 output (default settings, using the top 500 most variable genes). Significantly differentially expressed genes were intersected across treatments, and gene expression values were visualized using heatmaps representing the Z-score of relative expression (FPKM) per gene, using Microsoft Excel. Pearson correlation-based RNA-Seq sample clustering was performed in R (using the hclust package, complete linkage).

### 2.11 Functional enrichment

Functional annotations for genes were assigned using InterProScan v5.42 (Jones et al., 2014) (InterPro domains and Gene Ontology [GO]), GhostKOALA v2.2 (Kanehisa et al., 2016) (KEGG), SignalP v5.0 (Almagro Armenteros et al., 2019) (signal peptides and transmembrane domains) and SecretomeP v2.0 (Bendtsen et al., 2004) (non-classical secretion). Significant functional enrichment for GO terms was performed using GOSTATS v2.50 (Falcon and Gentleman, 2007) (adjusted P ≤ 0.05, minimum 3 genes differentially expressed) and for Interpro domains and KEGG pathways using WebGestalt v2019 (Liao et al., 2019). In order to test for significant enrichment of differentially expressed gene sets among previously published datasets, negative binomial distribution tests (P < 0.05 threshold) were ran for gene sets of interest containing at least 10 genes, and were compared to tissue-specific gene expression datasets (Rosa et al., 2014), intestinal compartment proteomics datasets (Rosa et al., 2015) and intestinal regions datasets (anterior, middle and posterior) (Gao et al., 2016). Previous dataset results were matched from the previous version of the *A. suum* genome (Jex et al., 2011) to the current version (Wang et al., 2017) by identifying top BLAST protein sequence similarity matches (E < 10^−5^, matches found for 77.6% of genes, of which 83.6% were bidirectional best hits).

### 2.12 Statistical tests

Differential expression and functional enrichment analysis (described above) all utilize FDR-based correction for multiple testing. Two-tailed T-tests to compare measurements of EGCN assumed unequal variance. For PI profiles, since all tests were performed with N = 20, significant positive detection of PI was determined to correspond to 25% PI detection (N = 5, P = 0.024), according to Fisher's exact test when control worms have zero PI detection. However, PI profiles were analyzed and compared qualitatively rather than requiring strict significance cutoffs.

## 3 Results

Frequently, whole-worm motility inhibition assays are used to evaluate effects of drugs and drug-like compounds on parasitic nematodes. Rapid resolution of individual cells and organs affected by these treatments in whole worms while in culture would greatly enhance details of likely relevance to the anthelmintic effects at play. In this investigation we integrate the use of i) fluorescent nuclear probes (bisbenzimide, propidium iodide), ii) a set of 6 small molecule inhibitors recently demonstrated to target the nematode intestine (nematode intestinal toxins/toxicants, NITs (Jasmer et al., 2020)), iii) an *Ascaris suum* larval parasite culture system and iv) RNA-seq transcriptional response to NITs, to conduct rapid pathologic and molecular assessment of NIT toxicity relative to many cells and organ systems in the whole worm. The approach was designed to discriminate performances among inhibitors in relation to their toxicity for cells and organ systems.

### 3.1 Fluorescent probes demonstrate larval ingestion and digestion

**Fig. 1:**
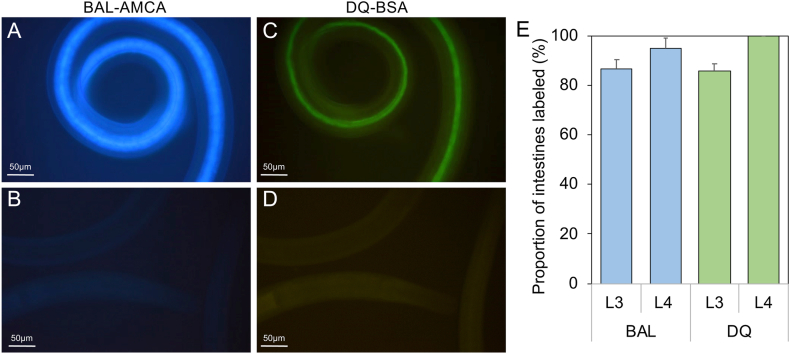
Ingestion of fluorescent probes by *A. suum* L3 and L4. L4 *A. suum* larvae stained with Beta-ala,lys-AMCA (BAL-AMCA) (**A**) or DQ-BSA (**C**) for 4 h and viewed for fluorescence. Unstained controls (**B, D**) respectively, showed no fluorescence signal, with corresponding epifluorescence filters. The proportion of L3 and L4 *A. suum* showing intestinal staining with BAL-AMCA (BAL) or DQ-BSA (DQ) (**E**).

To demonstrate larval feeding, we first assessed the ability to deliver fluorescent probes to the intestine of L3 and L4 *A. suum* in feeding assays using two function-based fluorescent probes: 1) beta-ala,lys-AMCA (BAL-AMCA), a fluorescent dipeptide that enters nematode intestinal cells by receptor mediated transport (Meissner et al., 2004); and DQ-BSA a protease substrate in which fluorescence quenched in the intact construct becomes visible upon proteolytic hydrolysis (Vazquez and Colombo, 2009). Feeding *A. suum* L3 and L4 with either of these fluorescent probes led to labeling of the intestinal lumen or cells in ≥86% of both larval stages within 4 h of exposure (Fig. 1), indicating competence of larvae to deliver these and other probes and treatments to the intestine via feeding. These results indicate the ability for larvae to ingest and/or subsequently digest the fluorescent probes, indicating possible introduction over the cuticular surface or apical intestinal membrane. Thus, evaluations can be performed to determine if membranes at these surfaces are permeable or compromised to allow entry into cells and body of these larvae.

### 3.2 Live *A. suum* larval nuclei localization using bisbenzimide (BB) labeling

**Fig. 2:**
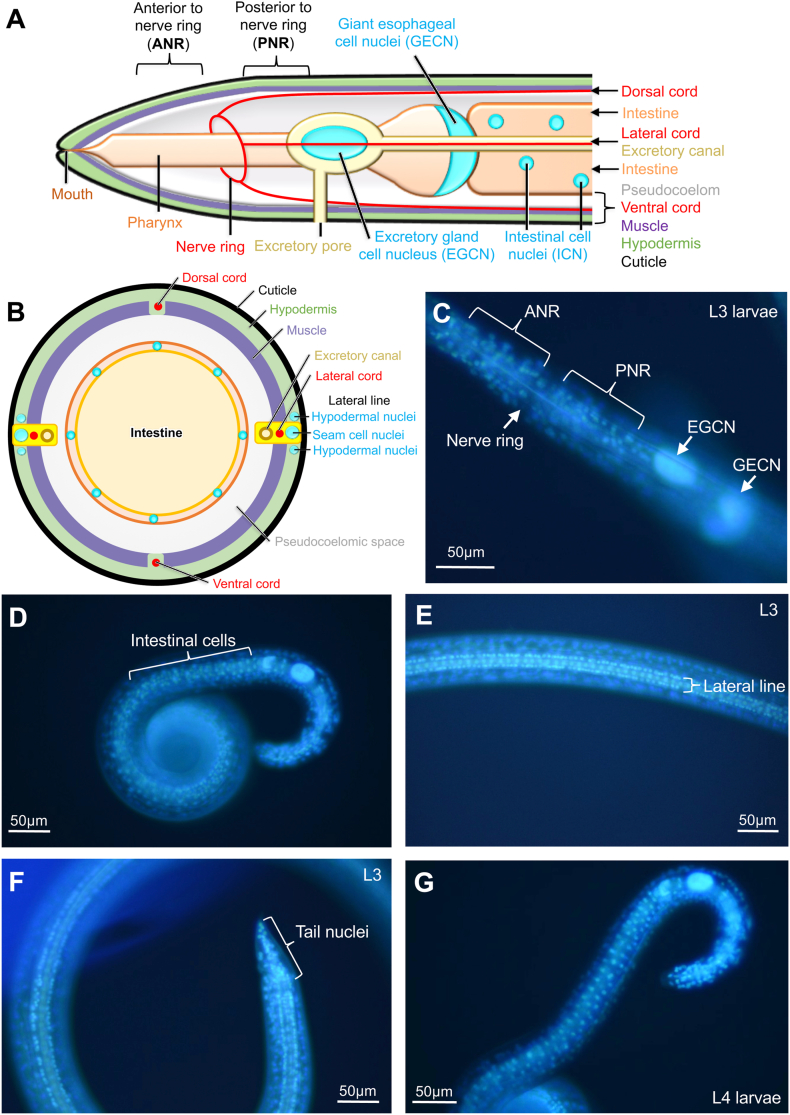
(**A**) Transverse and (**B**) cross-sectional diagrams of *A. suum* organs and nuclei positions. Bisbenzimide (BB) nuclei staining of L3 *A. suum* showing (**C**) nuclei and structures in the anterior end of the larvae, (**D**) intestinal cells posterior to the GECN, (**E**) the lateral line running longitudinally down the length of the larvae and (**F**) nuclei in the tail region. (**G**) L4 larva is shown for comparison and show very similar anatomy as outlined by nuclei.

As a baseline for monitoring cells and organs affected by toxic treatments, we labeled larvae with bisbenzimide (BB), a cell permeable DNA binding dye to establish a map of nuclei localization for various cells and/or organ systems within healthy larvae (Fig. 2A–B). This method labels nuclei of various cells and/or organ systems in *A. suum* larvae that can be resolved using a 20× microscope objective (Fig. 2C–F). We resolved nuclei in cells of various tissues, including intestinal cells (Fig. 2D), and the most prominent nucleus was observed is the excretory gland cell nucleus (EGCN), an apparent single large nucleus positioned anterior to the distal terminus of the esophagus and located medially in the lateral line superstructure, of which an excretory duct is a constituent element (Jenkins, 1970) (Fig. 2C). The next most prominent nuclei include a set of 3 giant esophageal cell nuclei (GECN) (Fig. 2C), located in cells at the intestinoesophageal junction of the tri-radiate esophagus. BB-labeled GECN most often appear as a U-shaped structure that accounts for two nuclei with a third nucleus at a different focal plane (*see* DIC results in the next section). Nuclei that comprise the lateral lines run nearly the full length of the body and consist of a longitudinal series of single file pairs of lateral and hypodermal cell nuclei with an intervening, central line of single seam cell nuclei (Fig. 2E). The nerve ring is typically composed of nerve fibers and routinely stands out as a circumesophageal region devoid of staining (Fig. 2C). Apparent neuronal nuclei (nerve cell bodies), from which nerve ring-fibers originate, localize both anterior and posterior to the nerve ring (Fig. 2C). Although easily resolved with DIC (see below), somatic muscle cell and esophageal cell nuclei other than GECN have proven problematic for observation with the current microscopic setup. Finally, prominent nuclei localized posterior to the anus (tail) likely include neuronal, muscle, hypodermal nuclei (Fig. 2F), as in other nematodes. Nuclei of L4 generally show the same level of resolution using BB (Fig. 2G).

### 3.3 Combining differential interference contrast microscopy (DIC) microscopy and BB labeling to increase resolution of *A. suum* larva nuclei localization

**Fig. 3:**
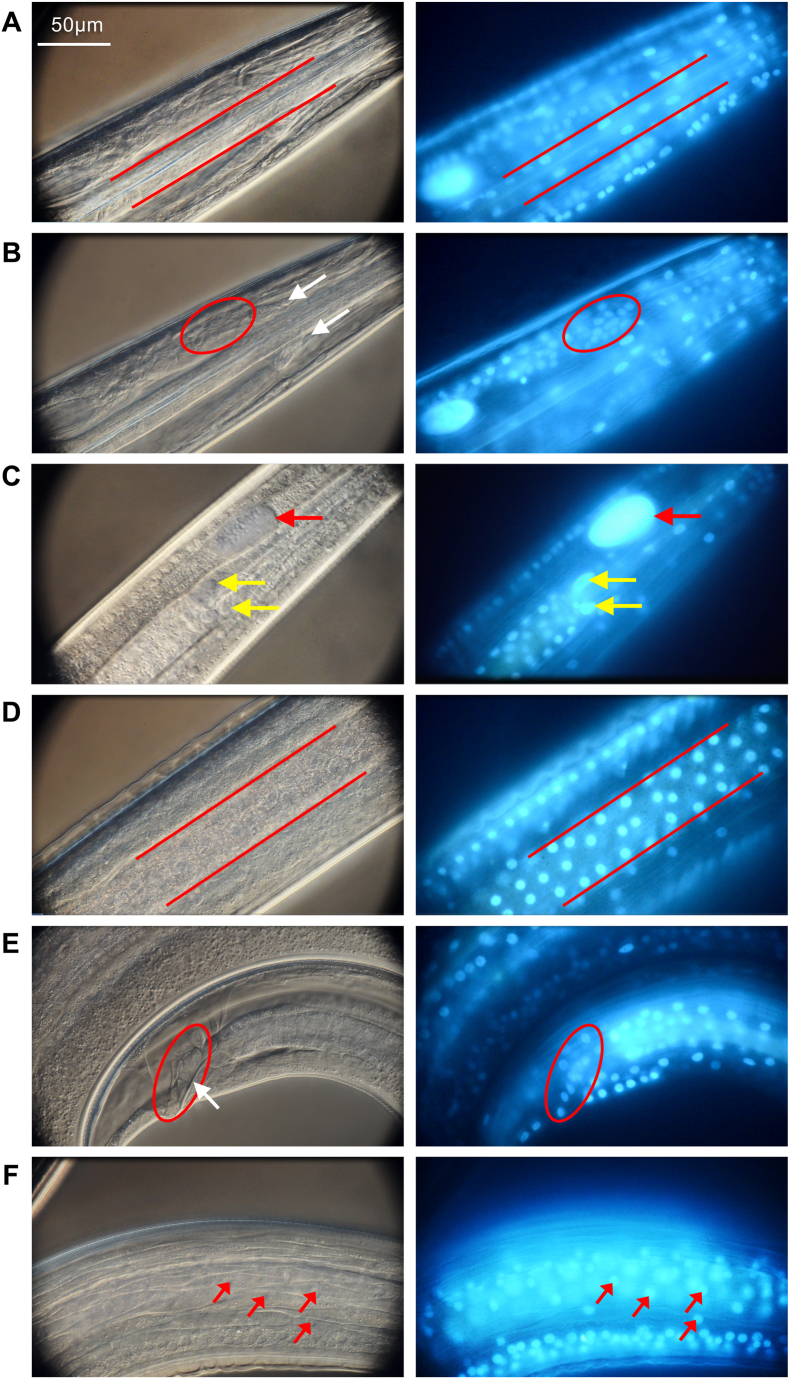
Differential interference contrast microscopy (DIC; left panels) and BB fluorescence (right panels) used in combination to distinguish *A. suum* L3 structures corresponding to (**A**) esophageal cell nuclei, (**B**) nerve cell nuclei (white arrows = nerve ring), (**C**) EGCN (red arrows) and GECN (yellow arrows), (**D**) middle-region intestine, (**E**) tail region posterior to anus (white arrow = anus), and (**F**) muscle cells. Scale shown in panel A applies to all panels. (For interpretation of the references to colour in this figure legend, the reader is referred to the Web version of this article.)

DIC (60× objective) coupled with BB fluorescence validated assignments of nuclei to organ systems made by using standard microscopic methods, and also resolved nuclei in somatic muscle and esophageal cells (Fig. 3). Thus, nuclei of cells comprising the esophagus, somatic musculature, EGCN, GECN, cells anterior and posterior to the nerve ring, intestinal cells, hypodermis, and cells posterior to the anus (tail), are all clearly resolved by this combination of microscopic methods. The individual components of DIC and fluorescence are complementary in that BB can localize nuclei that are difficult to resolve by DIC, or beyond the immediate focal plane of DIC resolution. Thus, apparently all, or nearly all cells, comprising larval *A. suum* can be resolved by methods utilizing BB coupled with standard and DIC microscopic methods in live worms. Although DIC offers greater resolution and allows us to establish and resolve baseline anatomy to better orient BB results, this method alone proved cumbersome and inefficient for assessment in experiments involving multiple treatments and even modest sample sizes of larva. Consequently, data for most experiments described below were obtained using a 20× objective (200× magnification) with an inverted, fluorescence microscope.

### 3.4 Evaluating NITs toxicity using propidium iodide (PI) labeling

**Fig. 4:**
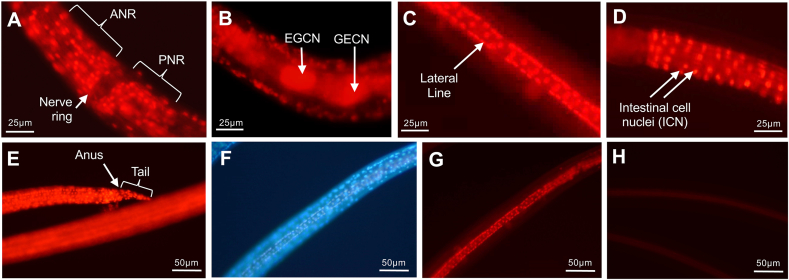
Cell death indicated by propidium iodide (PI) staining in nuclei of *A. suum* L4 larvae treated for 2 days with leflunomide (500 μM, A); staurosporine (25 μM, B,E); ruxolitinib (500 μM, C,F,G); alvocidib (500 μM, D). (**A**) Nuclei in the ANR and PNR, with no signal for the nerve ring, (**B**) the EGCN and GECN, (**C**) nuclei in the lateral line (hypodermis/seam cells), (**D**) intestinal cell nuclei (ICN) and (**E**) nuclei in the tail region, (**F**) Bisbenzimide and (**G**) PI staining of the middle regions of the same treated larva show exclusive PI labeling in the lateral line, revealed by the combination of the two stains. (**H**) Control larval nuclei were unlabeled by PI.

To evaluate the NITs’ toxicity, *A. suum* L4 were first allowed to ingest BB and PI for 4 h prior to addition of NITs (Fig. 4). BB was primarily used to discriminate between cells that exhibit detectable membrane damage (PI labeled nuclei) or not. Initial experiments included 6 different NITs in 48-h experiments and at concentrations that when combined were anticipated to provide positive results for the application of this assay (Jasmer et al., 2020). Whereas control larvae generally showed no PI staining of nuclei, weak fluorescence of free PI was detectable in the intestinal lumen, and BB staining confirmed the presence of nuclei with potential for PI staining (Fig. 4H). A small number of control L4 showed PI labeling of nuclei, which involved general staining of nuclei throughout the worm and occurred in L4 displaying damage from handling. In contrast, NIT treatments led to PI labeling of nuclei in many different cells and organ systems (Fig. 4A–E) including those anterior and posterior to the nerve ring, EGCN, GECN, intestine, lateral lines, and tail. With lower power objectives (20× and below), esophageal nuclei and somatic musculature were difficult to resolve. BB-staining illuminated nuclei that were either labeled or not with PI and often provided orientation for assignment of PI labeled nuclei to organ systems (Fig. 4F and G). PI was also found to emit weak red fluorescence when viewed using BB filters. Thus, nuclei co-labeled with PI and BB sometimes appeared pink (red and blue) when fluorescent filters to detect BB were used (Fig. 4F, some hypodermal and seam cell nuclei). These results show that PI staining can rapidly resolve, with unexpected breadth and clarity, cell damage consistent with cell death in many organs of *A. suum* L4.

### 3.5 Cell death profiles resolved by PI labeling

**Fig. 5:**
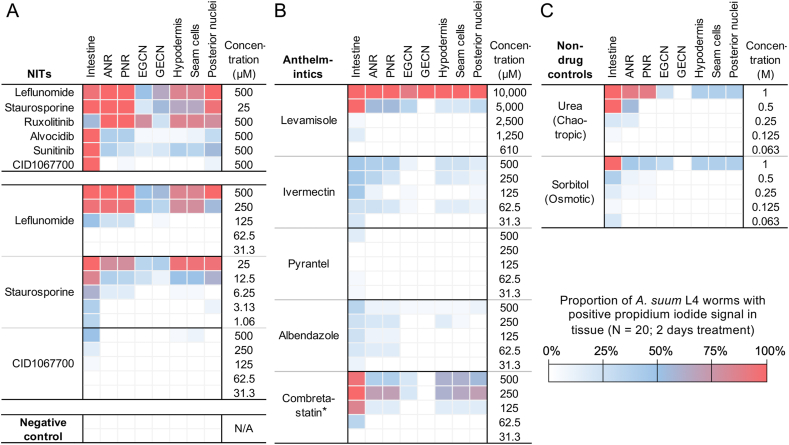
Propidium iodide signal observed in *A. suum* L4 worms in response to treatment with (**A**) NITs, (**B**) known anthelmintics (*excluding combretastatin, non-anthelmintic beta-tubulin inhibitor) and (**C**) non-drug controls.

Although PI staining of nuclei ensued in many organs with some NIT treatments, in other cases the distribution was observed to be more discriminating. To resolve this complexity, a classification scheme was designed based on a) signal in organ systems (Fig. 5A) (intestine, hypodermis, seam cells), b) specific nuclei (EGCN, GECN) or c) general location (anterior to nerve ring; posterior to nerve ring, but anterior to GECN, and exclusive of EGCN; tail, posterior to anus). PI staining was determined based on this scheme for the first 20 larvae encountered in each treatment. PI profiles show that different NITs produce different signatures of PI labeling and cell death. The percentages for each category provide assessment of variation according to each treatment. As an example, leflunomide consistently caused PI labeling to occur in multiple organs, cells and regions, although with some variation, as did staurosporine. In contrast, both alvocidib and CID1067700 effects were more, but not exclusively, restricted to intestinal cells, with CID1067700 producing the most intestinocentric profile. However, PI labeling tended to be patchy, rather than uniform, along the intestinal length for each of these two NITs. Ruxolitinib treatment caused significant, but moderate intestinal labeling, and the highest percentage of EGCN labeling. From these results PI labeling allows rapid in culture determination of cell death profiles that inform about a given NIT under the conditions described.

When profiles of all NITs are taken into account, nuclei in the intestinal cells and cells anterior and posterior to the nerve ring stand out as most commonly displaying PI labeling. The results then a) indicate a real potential of many specific cells and organs affected by NITs as targets in anthelmintic strategies, b) identify specific experimental treatments that confer toxicity to these cells and organs, and c) identify the intestine and cells anterior and posterior to the nerve ring as relatively sensitive to the NITs tested compared to other cells and organ systems assessed.

Dilutions were next conducted to determine concentration-dependent PI profiles for selected NITs (Fig. 5A). Leflunomide, staurosporine and CID1067700 were selected because leflunomide and staurosporine cause PI staining in nuclei of cells in all organ systems evaluated, whereas the IC50 for leflunomide is substantially higher than staurosporine (Jasmer et al., 2020), and PI staining conferred by CID1067700 treatment was restricted primarily to the intestine under conditions used here. In each case, the occurrence of PI nuclear staining was diminished among cells and organs with decreasing NIT concentration (Fig. 5A). Whereas treatment with CID1067700 primarily led to PI labeling in intestinal cells even at the highest doses, leflunomide and staurosporine showed PI staining restricted primarily to intestinal cells and cells anterior and posterior to the nerve ring at lower doses, thereby demonstrating relative sensitivities congruent with results from comparing PI labeling across all six NITs in the previous section.

### 3.6 PI profiles of several existing anthelmintics

We next sought to determine PI labeling profiles that might be generated by commercially available anthelmintics or related compounds, which have established major mechanisms of action, including impeding neurotransmission (ivermectin, pyrantel, levamisole) or binding to tubulin and depolymerizing microtubules (albendazole-SO). Dilution experiments were conducted with ivermectin, pyrantel, albendazole-SO starting at 500 μM to allow comparison to NITs, while levamisole was tested beginning at 10 mM because this concentration is used to anesthetize nematodes in vitro (Lockery et al., 2012; Patel and Soto, 2013). The non-anthelmintic combretastatin was included starting at 500 μM for comparison to albendazole-SO, the binding sites of both of which overlap at the colchicine site of beta-tubulin (Ehteda et al., 2013; Lu et al., 2020). Although not intended to address anthelmintic mechanisms *per se*, the range of concentrations tested for ivermectin, pyrantel and albendazole overlap with those tested on *A. suum* L4 in motility assays (Hu et al., 2013). With the exception of the minimal PI labeling profile produced by pyrantel, each of the other anthelmintics or compounds tested produced a robust PI labeling profile that encompassed one or more organ systems and diminished with decreasing concentrations, ultimately identifying the intestinal cells and cells anterior and posterior to the nerve ring as ones that stained at the lower drug concentrations (Fig. 5B). Thus, under the conditions of the experiment, which involve concentrations well above those expected for conferring anthelmintic effects in vivo and a single time point, toxic effects involving cell death were noted.

### 3.7 Physical factors that might affect PI labeling

We next tested effects conferred by NITs that may be independent of intended pharmacological effects (Fig. 5C). These include possible osmotic, chaotropic and pH effects, and the effects due to nutritional starvation.

*Osmotic and chaotropic effects*. The possibility that osmotic (hypertonic) or chaotropic effects of NITs could account for PI labeling profiles was tested in dose response to sorbitol (osmotic) or urea (osmotic and chaotropic) beginning at 1 M concentrations. At high concentrations, widespread nuclear PI labeling ensued, which rapidly decreased to negligible levels, with sparse PI labeling restricted to the intestine at the lower concentrations. Even considering unexpectedly high dissociation of NIT compounds in solution, the maximum NIT concentrations used (500 μM) are not expected to exceed 1–2 mOsm. Thus, neither osmotic nor chaotropic effects are sufficient to explain the PI labeling effects documented for the NITs. Nevertheless, relative sensitivities to these treatments could be ordered as intestinal cells > cells anterior and posterior to the nerve ring > hypodermal and seam cells, tail cells > other cells.

*A* pH *effect*. When the highest concentrations of NITs, urea and sorbitol used here were added to media, mean pH values taken immediately after removal from culture chambers were found to vary from approximately 7.4 (±0.10) to 7.79 (±09). We tested pH effects on larvae cultured from pH 7.46 to 7.74, which required change from a bicarbonate to HEPES (25 mM) buffered media to achieve the highest pH value. No effect leading to significant PI labeling of L4 was observed at any pH tested (Fig. 5C). Therefore, effects of pH do not explain the L4 PI labeling patterns induced by NITs in our experiments.

*A nutrient effect*. Because NIT treatments impair movement of larvae, we next examined the possibility that interference with nutrient uptake, potentially by inhibiting esophageal pumping, underlies pathology leading to PI labeling. L4 were cultured in PBS absent a nutrient source and then assessed for PI labeling. After two days in PBS culture no larvae displayed PI labeling, whereas after 5 days, 11 of 20 L4 showed low level labeling (sparse). In contrast, no labeling was observed at either treatment time point for media controls, and all larvae showed widespread labeling in the staurosporine positive controls on both assessment days. Consequently, although lack of nutrients can lead to minimal PI labeling by 5 days in culture, this level is insufficient to account for the PI labeling induced by NITs at the initial concentrations tested in assays reported above (2 days after treatment).

This series of experiments collectively dispel concerns that non-specific physical effects or indirect impedance of nutrient ingestion account for PI labeling induced by NITs. On the other hand, osmotic and/or chaotropic treatments led to PI nuclear staining, with intestinal cells and cells anterior and posterior to the nerve ring displaying the greatest sensitivity, albeit at concentrations orders of magnitude above those relevant to the NIT concentrations investigated here.

### 3.8 Toxic effects on the EGCN

**Fig. 6:**
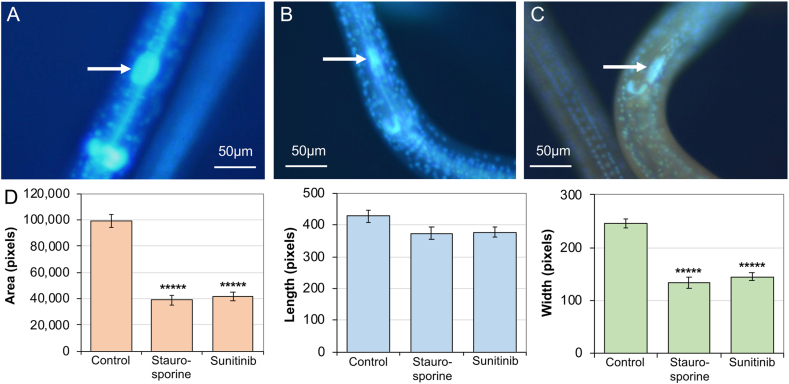
*A. suum* L4 larvae following two days of (**A**) Control conditions (**B**) Staurosporine treatment (25 μM) (**C**) Sunitinib treatment (500 μM). The EGCN (white arrows) shows altered morphology following drug treatment, with significant reduction in area, width but not length (**D**). Error bars represent standard error, ***** = P < 10^−7^ by two-tailed T test (unequal variance).

Independent of PI labeling, BB labeling of nuclei supported detection of altered nuclear morphology and abnormal spatial distributions of nuclei in intestinal tissue of unfixed whole larvae in culture following NIT treatments (Jasmer et al., 2020). Likewise, BB labeling aided visualization of altered nuclear morphology resulting from NIT treatments in other tissues. One of the best examples involves the large EGCN. This nucleus routinely showed atrophy after treatment with staurosporine and sunitinib (Fig. 6) as reflected by area when measured with lateral lines facing up and down. Mean EGCN area and width was significantly reduced compared to control larvae following treatments with both of these NITs. While length appeared to shorten, the effect was not significant (P > 0.05). Similar but more sporadic occurrence of EGCN atrophy was observed in relation to other NITs, but not to the extent observed with staurosporine and sunitinib. Although intensity of EGCN labeling suggests that change in DNA content might accompany atrophy in size, measurements here were compromised by background fluorescence conferred by both of these NITs. Nevertheless, the size of the EGCN is dramatically reduced by the two NIT treatments described. Incidentally, the GECN also show apparent atrophy as depicted in Fig. 6, but this was not quantified due to technical challenges related to shape and three dimensional topography of the three GECN.

In combination, our experiments demonstrate toxic effects of NITs on EGCN based on atrophy (BB labeling) and PI labeling, each fluorescent marker assessing different cellular properties. Consequently, we sought to assess relationships between EGCN atrophy and PI labeling. In a separate double labeling experiment, EGCN from ten staurosporine-treated or control L4 were assessed for EGCN area and PI labeling. All EGCN measured in the staurosporine treatment had areas that fell below the 95% confidence interval for control larvae. In contrast to the consistency of nuclear atrophy, only 4 out of 10 of the staurosporine-treated EGCN showed PI labeling, indicating cell death. Thus, cell death indicated by PI nuclear staining was observed in only a subset of EGCN that showed atrophy. This result may indicate independent processes, e.g. one leading to atrophy of the EGCN and another related to excretory cell membrane damage leading to PI labeling, or that atrophy precedes EGC death.

### 3.9 NIT effects on adult *A. suum*

**Fig. 7:**
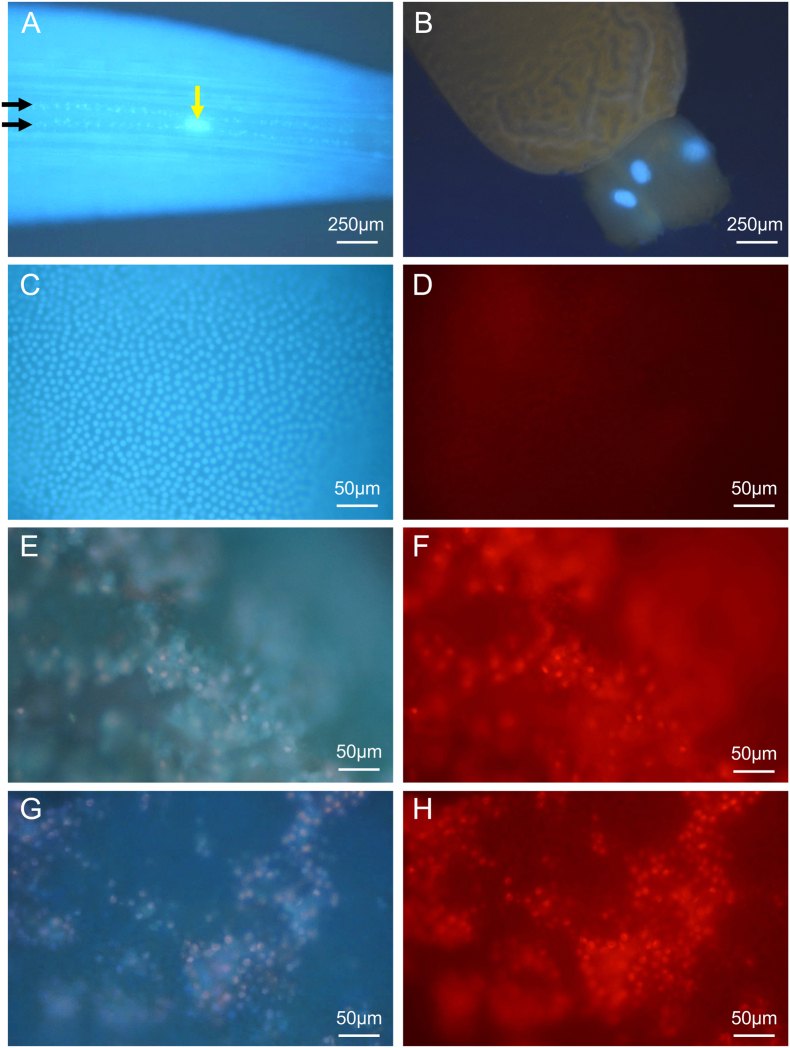
Bisbenzimide (BB) and propidium iodide (PI) staining in Adult *A. suum*. BB was injected into the pseudocoelom of normal adult female worms and nuclear staining was evaluated after 1 h to observe apparent EGCN (yellow arrow) in whole worm (**A**), GECN in dissected esophagus, (**B**), or intestinal nuclei in dissected intestine (**C)** (also representative of intestinal nuclei of experimental control worms cultured for 2 days in PBS). Dissected intestine from control worms cultured for two days in PBS displayed little to no PI staining (**D**). Dissected intestine from leflunomide (500 μM, **E**,**F**) or staurosporine (25 μM, **G**,**H**) treated (2 days), stained with BB and PI and viewed with UV-2A (blue, **E**,**G**) or G1A (red, **F**,**H**) epifluorescence filters. Black arrows in **A** identify hypodermal nuclei in the lateral line nuclei that straddle the large EGCN. (For interpretation of the references to colour in this figure legend, the reader is referred to the Web version of this article.)

BB and PI labeling methods were next used to assess the cytotoxicity of selected NITs in adult worms. Injection of BB into the pseudocoelom of adult *A. suum* produced rapid staining of nuclei after 1 h in several examples chosen to highlight the application (EGCN, GECN and intestinal nuclei; Fig. 7). We observed presumptive EGCN that were viewed in intact live worms using the inverted fluorescent microscope (4× objective). The presumptive EGCNs in question localize proximal to the esophagus and subtending the left lateral line nuclei and were approximately 40 μM long (Fig. 7A). Given that there are no other known nuclei of this size in this location, we conclude that these nuclei are EGCN. In contrast to L3 and L4, each GECN was clearly resolved in adults (Fig. 7B). BB staining of intestinal cells was uninterrupted (Fig. 7C), indicating comprehensive labeling of nuclei in this tissue, but only occasional PI labelling of the nuclei in untreated controls (Fig. 7D). Thus, staining with BB can be achieved with live adult *A. suum* tissues and in live worms, although viewing of labeled nuclei of most tissues will require dissection from adult worms. Next, two of the most effective NITs, leflunomide and staurosporine, were used to determine their efficacy on adult *A. suum* and their effects discernible in intestinal tissue with PI labeling. In adult *A. suum*, complete immotility was observed by day 2 for worms treated with leflunomide (4 experiments 2 worms each, inclusive of male and females) and with staurosporine (2 experiments, 2 worms each, inclusive of males and females). Matched controls for each experiment (2 male, or female) showed full movement at day 2. Intestine dissected from 2 day-treated worms and then incubated with dyes showed staining of extensive patches of nuclei after treatment with both NITs (Fig. 7E–H).

In some cases, patches of PI-labeled nuclei from NIT-treated worms were located adjacent to BB-labeled nuclei (apparent intact cells) that lacked PI staining. However, other areas of the intestinal tube, with an otherwise intact basal lamina, had PI-labeled patches of cell with no adjacent intact cells (no BB-only labeled nuclei), indicating denudation of intestinal cells from the basal lamina and more extensive tissue degeneration.

These results demonstrate the following: first, rapid high resolution of normal and damaged cells in unfixed tissues from adult *A. suum*; second, treatment with each of these NITs led to immotility of adult *A. suum* in culture; third, treatment with each NIT caused extensive damage to intestinal cells, inclusive of cell death, observable in whole dissected and unfixed intestine. These findings validate a new system involving treatments and experimental outcomes that can be used to investigate mechanisms by which NITs, and other treatments, cause irreparable damage to intestinal cells, and potentially other tissues.

### 3.10 Transcriptional response of *A. suum* L3 to selected NITs

**Fig. 8:**
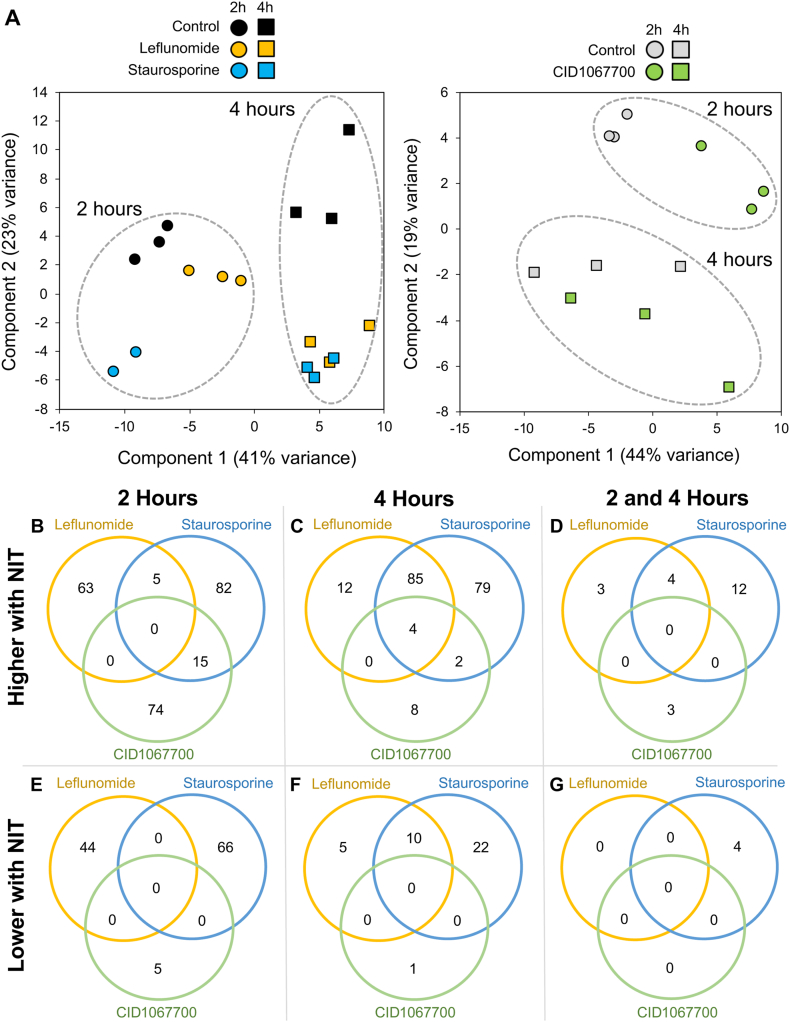
RNA-seq differential gene expression analysis. (**A**) Principal components analysis of RNA-Seq samples. (B–G) Significantly differentially expressed genes in response to NITs at 2 and 4 h, vs matched controls. Overlapping genes across the 3 NITs are shown for significantly upregulated genes in response to treatment after 2 h (**B**), 4 h (**C**), and both 2 and 4 h (**D**), as well as shown for significantly downregulated genes in response to treatment after 2 h (**E**), 4 h (**F**), and both 2 and 4 h (**G**).

NITs provide experimental reagents to induce cell death using the *A. suum* model described. Cell targets and pathways disrupted by NITs represent points at which cell death processes can be initiated, and thus can provide information of importance to general anthelmintic strategies. Gene response profiles induced by toxic treatments can inform about cell targets and pathways affected (Alexander-Dann et al., 2018; Pabon et al., 2018; Paananen and Fortino, 2019). Therefore, we used RNA-seq analysis to gain insight of this kind for three NITs selected for investigation here (leflunomide, staurosporine and CID1067700). Freshly isolated L3 were used in these experiments to minimize metabolic effects that might result from culture conditions, and each of the NITs have been shown to cause significant histopathology in L3 (Jasmer et al., 2020). RNA-seq was conducted for 2 and 4-h treatments to obtain relatively early gene response profiles. Samples were obtained in triplicate except for one staurosporine 2-h sample which failed due to technical reasons. Matched controls were also sequenced for each batch of samples collected. PCA analysis indicated low inter-replicate variability (Fig. 8A). Differential expression analysis (comparing each NIT treatment to its matched control) identified NIT-specific differentially expressed genes, as well as genes differentially expressed in response to more than one NIT (Fig. 8B–G). All gene annotations, read counts, normalized expression levels and differential expression statistics are available in Supplementary Table S2.

After 4 h, four genes were significantly upregulated by all three NIT treatments, including two *Ascaris*-specific proteins, a glutathione transferase (GST-7) ortholog and an NHR-3 ortholog (a nuclear hormone receptor transcription factor found in a *C. elegans* gene response profile to ethanol treatments (Kwon et al., 2004)). In *C. elegans*, GST-7 is a detoxification protein that is overexpressed by stress-induced phase 2 detoxification proteins, including in response to arsenite toxification (Crook-McMahon et al., 2014). A total of 85 genes were higher with leflunomide and staurosporine treatment after 4 h, and were significantly enriched for several gene ontology terms including “oxidoreductase activity” (P = 0.014) and “metal ion binding (P = 0.035). Among the most significantly enriched genes in these 85 were a carbonic anhydrase gene (one of which may function in detoxification of cyanate in *Ascaris lumbricoides* (Zolfaghari Emameh et al., 2015)) and a dehydrogenase 7 (DHS-7) ortholog, which was one of five genes also upregulated at 2 h with both drugs. In both *C. elegans* and *Haemonchus contortus*, several dehydrogenases (dhs-23, dhs-27 and dhrs7b) were among 41 genes commonly upregulated in response to five different benzimidazole drugs (Stasiuk et al., 2019).

### 3.11 NIT-specific differentially expressed genes and enriched pathways

**Fig. 9:**
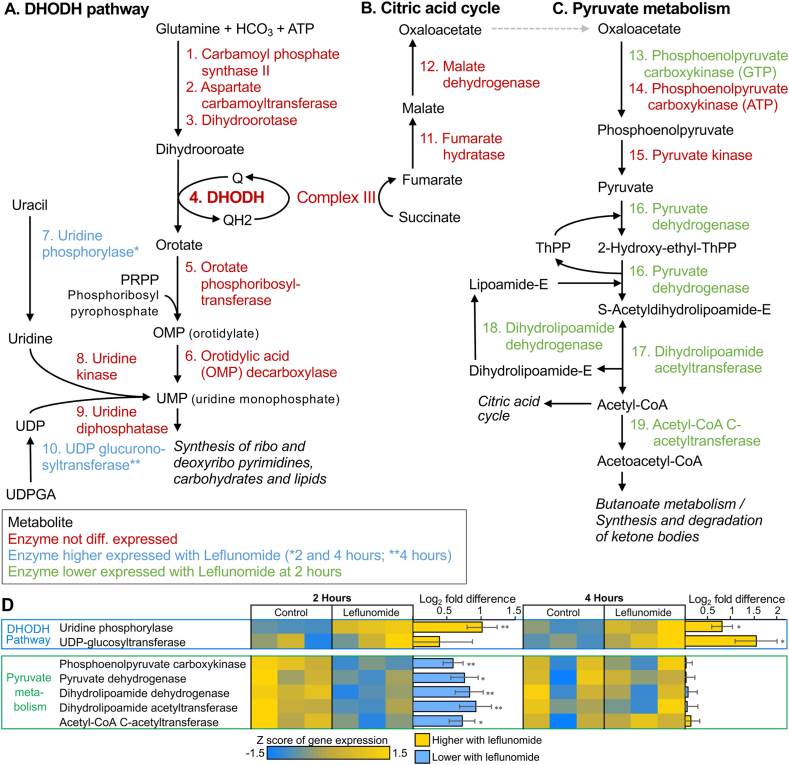
DHODH-associated pathways containing genes differentially expressed by leflunomide exposure.

More genes were significantly differentially expressed exclusively by leflunomide at 2 h (63 higher, 44 lower) than at 4 h (12 higher, 5 lower). Only 3 genes were significant at both 2 and 4 h, including: (i) a NEP-1 ortholog (neprilysin; K08635), which in *C. elegans* is required for locomotion and pharyngeal pumping (Spanier et al., 2005), (ii) a cation efflux protein and (iii) the *A. suum* UPP-1 ortholog (uridine phosphorylase; K00757). In humans, leflunomide targets only dihydroorotate dehydrogenase (DHODH) at lower doses, with some inhibition of tyrosine kinases at higher doses (Breedveld and Dayer, 2000). The UPP enzyme converts uracil to uridine, which can convert to UMP, an important downstream product of DHODH that is reduced when DHODH is inhibited (Greene et al., 1995). Additionally, at 4 h, UDP glucuronosyltransferase is also upregulated by leflunomide treatment, also resulting in increased UMP production (Fig. 9A), possibly compensating for DHODH loss. In human cell cultures, supplementation with uridine rescues the phenotype resulting from DHODH inhibition (Koundinya et al., 2018). The most significant (P = 6.3 × 10^−4^) of the 18 KEGG pathways significantly enriched among the 44 genes lower with leflunomide at 2 h (Supplementary Table S3) was “pyruvate metabolism”, which is also potentially affected downstream of DHODH activity, through its effects on the citric acid cycle (limiting oxaloacetate Fig. 9B, which is required for pyruvate metabolism; Fig. 9C). Within this pathway, all of the downregulated genes are immediately downstream from oxaloacetate, which is a product of DHODH activity. Together, these results are consistent with leflunomide inhibition of the predicted target, DHODH, and that the differentially expressed genes suggest pathway compensation for the loss of its activity.

CID1067700 has been identified as a Rab GTPase inhibitor (Aits et al., 2015). Rabs direct the trafficking of cargo from endosomes and autophagosomes along microtubules and participates in the fusion step with lysosomes (Hyttinen et al., 2013), and also coordinate cytoskeletal organization through interactions with myosin (Borg et al., 2014). After 2 h, the only significant KEGG pathway enriched among the 74 genes only upregulated by CID1067700 was “cytoskeleton proteins” (P = 0.017), and included three myosin genes, troponin T, a tubulin gene and a plectin gene. These results present evidence that Rabs are a potential target for CID106770 in nematodes.

Staurosporine is a more general kinase inhibitor and thus present a greater challenge in pinpointing initiator events that can lead to cell death in *A. suum*. However the gene response profile induced by staurosporine may provide information on the terminal process of cell death. In human cell lines, staurosporine is known to induce apoptosis via caspase-9 activation in the intrinsic apoptosis signaling pathway, independent of Apaf-1 and apoptosome formation (Manns et al., 2011; Malsy et al., 2019). Activation of this pathway is characterized by lysosomal membrane permeabilization and the release of cathepsin proteases into the cytoplasm (Wang et al., 2018), which also overlaps with elements of necrosis. Among the 82 genes higher only with staurosporine after 2 h, the only significantly enriched KEGG pathway was “Lysosome (4142)” (P = 0.0011), represented by 3 cathepsin genes (C, D and F), two SLC17A5 orthologs, and LITAF (required for activation of apoptosis in human cell lines (Shi et al., 2016)). Among the 22 genes lower only with staurosporine at 4 h, there is significant enrichment for genes containing Epidermal Growth Factor (EGF)-like domains (IPR000152, P = 2.8 × 10^−4^; Supplementary Table S3). In mice, EGF interactions with EGFRs protect against intestinal cell apoptosis induced by TNF (Yamaoka et al., 2008). Taken together, these results provide specific information on cell components that are induced by staurosporine and might mediate the terminal cell death process.

## 4 Discussion

This study presents an interdisciplinary approach designed to intersect i) technical advances that comprehensively and rapidly resolved in whole unfixed parasites pathologic damage induced by NITs among most cells and organ systems that comprise life cycle stages of *A. suum*, and ii) interrogation of transcriptional profiles resulting in identification of hypothetical targets of the drug-like compounds. The assays directly identify the constellation of cells and organ systems that undergo cell death with respect to each of multiple toxins/toxicants. Susceptibility to inducible cell death adds rationale to investigate the cells and organs identified in anthelmintic research, and advances established here provide new experimental methods to do so. We also identify, for some NITs, cellular pathways and molecular targets that are potential antecedents to irreparable pathologic outcomes. Our original derivation of NITs involved a *de novo* approach (Jasmer et al., 2020), which when integrated with methods and results described here establish a unique and useful experimental system for the purposes of discovery and detailed analysis of anthelmintic compounds and investigation of mechanisms of their toxicity. Ultimately, knowledge of mechanisms that actually mediate cell death coupled with pathways whose disruption initiate death processes hold much potential for application in anthelmintic research. Our results represent an important step in this direction.

There is no other in culture whole-worm assessment with comparable cellular resolution of cell death for parasitic nematodes. 10.13039/100006150PI labeling routinely supports monitoring of viability in many tissue culture cell types (Brana et al., 2002; Zhao et al., 2010; Cummings et al., 2012) and has been used with intestinal cells of the non-parasitic *C. elegans* relative to ingested toxins and otherwise intestinocentric pathology (Zhang et al., 2016). General body PI signal was also used to assess heavy metal toxicity in *C. elegans*, but without clear documentation of cells or organs affected (Hunt et al., 2012). However, it is the clarity, interpretability and relatively comprehensive whole worm assessment provided by the PI/BB method described here with *A. suum* that constitutes the uniqueness of our findings. The application also applies to adult *A. suum*. The detail provided is different from other microscopic methods (histology and electron microscopy) and can rapidly focus research on specific organs, cells, and potentially molecular targets and mechanisms of pathology for investigation by other approaches. When coupled with DIC, and presumably confocal microscopy, greater detail is attainable, although at a cost to efficiency of assessments. The detail obtained at lower magnification with less sophisticated methods identifies a system with substantial versatility to meet a range of research goals.

A caveat in interpreting PI labeling is that it provides assessment of cells to which PI is accessible. General body distribution of PI is likely to occur if it gains access to the pseudocoelomic fluid, which bathes most or all organs and cells of the nematode body. Breaching by PI of the internal gut (inclusive of the single cell thick intestinal tube) layer which is continuous with the outside environment, or the external cuticular-hypodermal-muscular layers, might be sufficient to accomplish this access. In this vein, the ability to deliver PI to the intestine pre-treatment is likely to increase sensitivity of the assay. Under conditions of positive PI signal, the assay has value for identifying affected cells and to conduct comparisons among experimental treatments. The interpretation of negative staining may vary, however, according to cell location. Lack of PI staining in cells at external (hypodermis) or internal (intestine) surfaces likely indicates cell viability, if ingested PI is available at the internal surface. In contrast, cells that die in internal organs, absent access of PI could go unnoticed in this assay. For dissected tissues, as with adult *A. suum*, the issue of PI access is markedly reduced or eliminated.

As another caveate, we chose a single time point to assess treatment effects here in order to demonstrate utility of the method. Longer exposure may demonstrate cell death for lower treatment concentrations at which this outcome was not observed at 2 days. However the optimal time course will likely depend on both concentration and the specific toxin/toxicant under investigation. Additionally, although PI labeling provided for objective determination of nuclear labeling, subjectivity in scoring among the categories of cell, organs and regions varied to some extent, as might be expected, and consultation with BB labeling proved helpful in making assignments. Also, variation within treatments is reflected by percentage labeling for each category of cell, organ, or region assessed, rather than quantitative assessment of numbers of cells (at least hundreds for each larva), which would have made the analysis far too cumbersome. Consequently, non-parametric measures made sense for feasibility of this analyses. Nevertheless, the approach was adapted with these caveats in mind and addressed the specific questions under investigation in this study.

PI labeling produced different cell and organ profiles for different NITs and other treatments, which indicates properties of individual NITs that may have bearing on mechanisms of action. For example, leflunomide and staurosporine produced the most inclusive PI labeling across cells and organ systems, suggesting inhibition of processes by these NITs that lead to cell death in many cells and organs. In contrast, CID1067700 and alvocidib produced more intestinocentric labeling, which may indicate inhibition of target/pathways preferentially expressed in intestinal cells, or a greater sensitivity of intestinal cells once inhibition is achieved. Additionally, BB staining of the EGCN identified morphologic atrophy that was most pronounced for sunitinib and staurosporine treatments. When coupled with knowledge of predicted or actual cellular targets, perhaps contributed by RNA-seq data (see below), knowledge of specific cells affected may aid in dissection of mechanisms involved.

Overall comparisons among the different NITs and anthelmintics tested identified cells located in the intestine and adjacent to the nerve ring as most frequently showing PI staining. Reports that intestinal cells are relatively sensitive to benzimidazole anthelmintics in some, but not all (O'Neill et al., 2016), parasitic nematodes might be explained by the specific mechanism of inhibition, e.g. depolymerization of microtubules. However, the more general sensitivity detected here suggests a more general explanation, which could involve location at an interface where high concentrations of toxic compounds are encountered, or intestinal cell characteristics not yet understood. In contrast to intestinal cells, relative sensitivity to toxic treatments has not been ascribed to presumptive neurons adjacent to the nerve ring. By comparison to toxicity that inhibits neurotransmission at receptor levels, our results indicate cell degenerative effects, which presumably is required for PI labeling to occur. Hypersensitivity leading to cell death in any crucial cell population is an important finding relative to anthelmintic research. Our results have validated (e.g. intestinal cells) and newly identified (e.g. putative neurons adjacent to the nerve ring) biologically distinct cell populations that each warrant investigation in this regard.

The PI profiles produced by existing anthelmintics was compared with NITs. Hence, the concentrations used well exceeded those recommended for therapeutic applications, and PI labeling showed dose dependent effects that were extinguished at lower concentrations. While the results may or may not relate to actual mechanisms of these anthelmintics, the PI profiles generated provide information that can be integrated into more complete investigations of these anthelmintics. The possibility that additional exposure time (>48 h) would have produced significant PI signatures at some of the lower concentrations also cannot be excluded. In contrast to the neurotoxic anthelmintics, benzimidazole microtubule inhibitors are known to cause cell degeneration in nematodes. In this regard, the broad range of cells demonstrating PI labeling in *A. suum* L4 induced by albendazole-SO are relevant. Combretastatin is not an approved anthelmintic, and although having distinct contacts, it also binds to the colchicine binding site of beta-tubulin (Gaspari et al., 2017), as do benzimidazole anthelmintics. Combretastatin conferred PI labeling that was similar to but more extensive than albendazole-SO. Accordingly, comparison of EC-50 values on motility (Jasmer et al., 2020) suggest somewhat better performance of combretastatin, which may relate to the comparative PI and dilution profiles generated here. Thus, PI labeling signatures may provide comparative information between, or among, different anthelmintics/NITs with related molecular targets. It seems reasonable to project that this monitoring system also has potential application to investigations on anthelmintic resistance in parasitic nematodes.

PI labeling indicates irreparable cellular damage and cell death, and apoptosis and necrosis are two major mechanisms known to mediate cell death in nematodes (Coburn et al., 2013). Two significant features of each mechanism are that they are latent and can be manipulated pharmacologically (Oh et al., 2003; Green and Kroemer, 2005). Therein lies a value for determining how NITs, with their diverse targets, can each initiate a process leading to *A. suum* cell death. As such, toxic compounds that initiate processes leading to cell death provide tools to investigate mechanisms of importance in anthelmintic research.

In context of initiator pathways, early gene expression responses to leflunomide treatment generated the greatest insight here and identified DHODH and uridine synthesis pathway inhibition as a possible entry point into a process leading to cell death. Although other targets and pathways may contribute to the leflunomide effects, the effects on genes in the primary uridine synthesis pathway, and then secondary energy generating pathways, was remarkably consistent with predictions for DHODH as a target for leflunomide. CID1067700 is a RAB GTPase inhibitor, and gene responses to this treatment differed from that of leflunomide, demonstrating specificity, and included cytoskeletal components that may reflect disruption of vesicular transport pathways. The strength of these findings is somewhat muted by limited information on pathways that respond to RAB GTPase inhibition in other organisms and cells. Nevertheless, inhibition of RAB GTPases and disruption of vesicular transport pathways may represent initiators of processes leading to cell death in *A. suum*. Support for this general view comes from the irreparable damage produced in intestinal cells of *A. suum* and *Haemonchus contortus* by benzimidazole treatments, which also disrupted intestinal vesicle transport (Borgers and De Nollin, 1975; Borgers et al., 1975; Jasmer et al., 2000; O'Neill et al., 2015). Despite the large number of genes impacted by staurosporine, a dominant pathway affected was not identifiable most likely due to the broad kinase specificity of this NIT. It may be significant that lysosomal enzyme genes stood out as activated by this treatment, and lysosomal hydrolases are important mediators of necrosis (Boya and Kroemer, 2008; Wang et al., 2018). It is possible that this response to staurosporine potentiates lysosomal contributions to cell death. Our results indicate that there are multiple early entry points to initiate processes that each lead to cell death in many cells and organs of *A. suum*. Nevertheless, these results provide a first glimpse of processes that may converge on this outcome. Many other cellular changes likely take place in the time span between RNAseq (2 and 4 h) and cell death assessments (48 h), and likely comprise a complex picture to untangle. Overall, the system described offers tools to investigate changes that unfold later, as well as acquire potentially insightful information regardinng *A. suum* cellular targets and pathways affected by other NITs.

Finally, we identified four genes that were upregulated by all three NITs investigated, which are of interest because they may reflect a common response to stress. In this context, 2 of the 4 genes encode glutathione S transferase and NHR-3, homologues of both of which are tied to stress responses in *C. elegans*. Because inhibitors of stress response proteins can enhance effects of anthelmintics, including parasitic nematodes, it will be of interest to determine if this gene response is common to other toxic perturbations in *A. suum* and other parasitic nematodes.

## 5 Conclusion

In this study we made technical advances that rapidly and comprehensively resolved pathologic damage induced by NITs among most cells and organ systems that comprise the *A. suum* parasite. This advance was complemented with gene response profiles, resulting in the identification of hypothetical targets of the NITs and cell pathways impacted by treatments that may reflect initiators of cell death processes. There is no other in culture whole-worm assessment with comparable cellular resolution of pathology for parasitic nematodes, thus the approach resulted in significant findings, especially because it implies that the pathological and molecular patterns are NIT-specific. The observed discriminatory nature of the profiles has broad applicability, including the identification of drugs able to induce similar pathologies, thus potentially having similar targets and/or mechanisms as well as identifying similar pathologies and molecular responses of new small molecule inhibitors. The results support the overall approach to have high value toward identifying cells and pathways that, when perturbed, can initiate processes leading to cell death in nematode cells.

## Declaration of competing interest

The authors declare that they have no known competing financial interests or personal relationships that could have appeared to influence the work reported in this paper.

## Glossary of Abbreviations

BAL-AMCA: beta-ala,lys-AMCA
BB: Bisbenzimide
DIC: Differential interference contrast
DHODH: Dihydroorotate dehydrogenase
EGCN: Excretory gland cell nucleus
GECN: Giant esophageal cell nuclei
LED: Larvae ensheathed with deformation
NIT: Nematode intestinal toxins/toxicants
PI: Propidium iodide
